# Artemisitene Alters LPS-Induced Oxidative stress, inflammation and Ferroptosis in Liver Through Nrf2/HO-1 and NF-kB Pathway

**DOI:** 10.3389/fphar.2023.1177542

**Published:** 2023-04-25

**Authors:** Changzhi Zhao, Congshu Xiao, Songqiao Feng, Jianxin Bai

**Affiliations:** ^1^ Second Affiliated Hospital of Dalian Medical University, Dalian, China; ^2^ Dalian Municipal Friendship Hospital, Dalian, China

**Keywords:** liver sepsis, ferroptosis, Nrf2, artemisitene, NF-κB

## Abstract

The liver plays a critical role in sepsis, which is a serious worldwide public health problem. A novel mechanism of controlled cell death called ferroptosis has recently been described. Disrupted redox equilibrium, excessive iron, and enhanced lipid peroxidation are key features of ferroptosis. It is unknown how ferroptosis affects liver damage caused by sepsis. In the present study, we aimed to elucidate the pathways and explore the impact of artemisitene (ATT) on ferroptosis in sepsis-induced liver injury. Our findings demonstrated that ATT significantly decreased liver damage and ferroptotic characteristics. Additionally, ATT significantly reduced the expression of the nuclear factor-κB (NF-κB) subunit to reduce LPS-induced hepatic oxidative stress and inflammation and upregulated the expression of nuclear factor-erythroid 2 (NF-E2)-related factor 2 (Nrf2) and its downstream protein heme oxygenase 1 (HO-1). This may offer a new strategy for preventing LPS-induced hepatic injury.

## Introduction

In the critical care unit, sepsis is one of the most frequent causes of death for hospitalized patients. This condition results in potentially fatal organ failure caused by an unbalanced host response to infection and represents a significant global public health concern (Rello et al., 2017). Through mechanisms such as bacterial clearance, the synthesis of acute-phase proteins or cytokines, and metabolic adaptation to inflammation, the liver plays a crucial role in sepsis and is crucial to controlling immune defense during systemic infections (Li et al., 2021). Determining the mechanism of liver cell death would be helpful for treating sepsis.

Stockwell originally described ferroptosis in 2012: a novel, intentional method of cell death that is iron-dependent and distinct from necrosis, apoptosis, and autophagy (Li et al., 2022). Because lipid peroxides cannot be metabolized by the GPX4-catalyzed reduction reaction and Fe^2+^ oxidizes lipids in a Fenton-like manner, a significant amount of reactive oxygen species (ROS) is produced, which induces ferroptosis (Su et al., 2019). Intracellular glutathione (GSH) depletion and decreased glutathione peroxidase 4 (GPX4) activity are the main biochemical causes of ferroptosis (Dixon et al., 2012; Lachaier et al., 2014; Stockwell et al., 2017). Many studies have demonstrated the tight connection between ferroptosis and the pathophysiological mechanisms behind an increasing number of diseases, such as tumors, neurological disorders, ischemia and reperfusion injury, hematological disorders, and renal damage (Martin-Sanchez et al., 2017; Adedoyin et al., 2018; Li et al., 2019; Fang et al., 2020). Iron is mostly stored in the liver, and liver disease is directly associated with iron overload (Yang et al., 2022). Recent research showed that the upstream factor in the acceleration of ferroptosis in liver fibrosis was the suppression of the tumor suppressor P53 (Wang et al., 2019). Few studies, however, have highlighted the importance of ferroptosis in sepsis-induced liver damage. However, inhibiting ferroptosis could represent a cutting-edge therapeutic strategy for liver damage caused by sepsis.

The redox-sensitive transcription factor nuclear factor-erythroid 2 (NF-E2)-related factor 2 (Nrf2) stimulates the transcription of antioxidant, cytoprotective, and anti-inflammatory genes (Wang X. et al., 2022). During normal conditions, Nrf2 is bound to its antagonistic regulator Kelch-like ECH-associated protein 1 (Keap1), which ubiquitylates Nrf2 and causes its degradation (Gu et al., 2017). In response to result of oxidative stress, Nrf2 dissociates from Keap1 and enters the nucleus. When Nrf2 and the small Maf proteins heterodimerize, antioxidant response elements (AREs) in the promoter regions of the target genes of Nrf2 are activated in the nucleus (He et al., 2020). Recent research has revealed that Nrf2 activation may inhibit the ferritinophagy pathway, inhibiting the ferroptotic response caused by lipid peroxidation in chronic obstructive pulmonary illness caused by PM2.5 (Guo et al., 2022). Nuclear factor-κB subunit (NF-κB) expression can be decreased by Nrf2 overexpression to reduce inflammatory responses. Artemisitene (ATT) is a byproduct of the antimalarial medication artemisinin and is also present in the herb sweet wormwood (Chen et al., 2018). ATT is an antioxidant and anticancer agent that activates Nrf2. ATT stimulates Nrf2 by decreasing Nrf2 ubiquitination and increasing its stability (Chen et al., 2016). Few studies have examined how ATT influences ferroptosis in LPS-induced liver injury. In the current study, we investigated the effects of ATT on the ferroptotic response *in vivo* and *in vitro* to develop a novel preventive approach for liver damage caused by sepsis.

## Methods

### Animals and treatment

Eight-to ten-week-old (∼24 g) male C57BL/6 mice were kept in a 12 h light–dark cycle setting with free access to food and water. We purchased ATT from Med Chem Express (HY-122550). Four groups of mice were created: control, ATT, LPS, and LPS + ATT (*n* = 25 in each group). LPS (20 mg/kg) was injected intraperitoneally to create a sepsis model for 24 h (Zhang et al., 2022). In the control group, sterile saline was injected. Two hours prior to the injection of LPS, the LPS + ATT groups received intraperitoneal injections of ATT (10 mg/kg) every 4 h (Chen et al., 2016). Blood and liver tissues from five anesthetized mice were collected. To ascertain the survival rate and changes in body weight, the other 20 mice were fed for 5 days. The Dalian Medical University Institutional Animal Care and Use Committee approved each step involved in the animal study.

### Histologic analysis

Formalin was used to fix liver tissue before paraffin embedding or the use of optimal cutting temperature compound (OCT). Prussian blue staining and hematoxylin and eosin (H&E) staining were performed. The evaluation of the liver histological score involved the quantitative measurement of tissue damage under a light microscope by a blinded observer. The total of the individual scores for each of the following six items—cytoplasmic color fading, vacuolization, nuclear condensation, nuclear fragmentation, nuclear fading, and erythrocyte stasis—ranged from 0 to 18 and was used to calculate the liver histological score (Bi et al., 2019).

### Liver function and damage quantification

To determine liver function, serum samples were collected. By measuring the levels of aspartate aminotransferase (AST, Cat# C010-1-1, Nanjing Jiancheng Bioengineering Institute) and alanine aminotransferase (ALT, Cat# C009-1-1, Nanjing Jiancheng Bioengineering Institute), liver function was calculated. The Second Affiliated Hospital of Dalian Medical University used an automated approach to assess the levels of AST and ALT.

### Measurements of GSH, SOD, and MDA levels

According to the manufacturer’s instructions, MDA, SOD, and GSH kits (Beyotime Biotechnology) were used to measure the levels of MDA, SOD, and GSH in liver tissues and cells.

### Iron assay

According to the product protocol, the amount of iron in serum and cell culture plates was determined using an iron assay kit (#ab83366, Abcam).

### Cell culture and experiments

The L-02 cell line was made available by Dalian Medical University. Cells were grown in 1,640 supplemented with 10% fetal bovine serum and antibiotics (100 U/mL penicillin and 100 μg/mL streptomycin, Sigma). The cells were cultured at 37°C in a humidified environment with 5% CO^2^. Cells were pretreated with ATT (2 μM), ferrostatin-1 (Fer-1, 10 μM) or Nrf2 inhibitor - Ml385 (1 μM) for 1 h and then LPS (1 μg/mL) was added (Chen W et al., 2016; Wang Z. et al., 2022; Yang W et al., 2022).

### Cell viability assay

Cell viability was evaluated using the MTT assay. First, 1 × 10^3^ L-02 cells in the logarithmic growth phase were plated in 96-well plates. After 24 h, the drugs were administered. At specific times, MTT reagent (10 μL/well) was added to the wells. After 4 h, dimethyl sulfoxide (DMSO) was added, and the plate was agitated for 20 min at 37°C. The absorbance at 570 nm was then measured using a microplate reader.

### Dihydroethidium

To measure the amount of ROS in livers and L-02 cells, dihydroethidium (DHE) was used. DHE (10 μM) was applied to OCT liver slices (8 μm) at 37°C for 30 min. L-02 cells were first plated in 6-well culture plates for 24 h, followed by 8 h of ATT treatment at a concentration of 2 μM and 24 h of LPS treatment (Chen et al., 2016). Cells in the LPS group were cultured without ATT pretreatment, while cells in the control group were cultured normally in RPMI 1640 without serum. DHE (10 μM) was added to the cells. Images were then obtained using fluorescence microscopy (Nikon, Japan) with a ×200 overall magnification after the cells had been rinsed three times with serum-free 1,640.

### Western blot assay

Total proteins were extracted from snap-frozen liver tissues or cells with a protein extraction kit (Keygenbio, KGP250) and centrifuge tube (Guangzhou Jet Bio-Filtration Co., Ltd.). The protein lysates (25 μg) were electrophoretically separated on a 10%–12% SDS‒PAGE gel before being transferred to polyvinylidene difluoride (PVDF) membranes. The blots were treated with the proper antibodies at 4°C overnight, followed by incubation with a goat anti-rabbit or mouse secondary antibody (Sino Biological Inc.). The blots were processed with an ECL Plus chemiluminescence system. Anti-Nrf2 (WL02135, Wanleibio), anti-Lamin B (WL01775, Wanleibio), anti-heme oxygenase 1 (HO-1, ab68477, Abcam), anti-P65 (#HA500402, HUABIO), anti-phospho-P65 (p-P65, #3039, Cell Signaling Technologies), and anti-GPX4 (#ER 1803-15, HUABIO) antibodies were used. Densitometry was performed using ImageJ software, and GAPDH (#ER1706-83, HUABIO) was used as an internal control.

### Real-time PCR

We purified total RNA from fresh livers and cells using TRIzol reagent (Invitrogen, New York) in accordance with the manufacturer’s instructions. Using a Superscript II kit, first-strand cDNA (2 μg) was created (TAKARA, Japan). Sangon Biotech Corporation synthesized the primers (Shanghai, China). Table 1 contains the primer sequences.

**TABLE 1:** Primers used for qPCR.

Gene	Forward primer (5′-3′)	Reverse primer (5′-3′)
IL-1β	TGC​CAC​CTT​TTG​ACA​GTG​ATG	TGA​TGT​GCT​GCT​GCG​AGA​TT
TNF-α	CAG​GCG​GTG​CCT​ATG​TCT​C	CGA​TCA​CCC​CGA​AGT​TCA​GTA​G
IL-6	TGA​TGG​ATG​CTA​CCA​AAC​TGG​A	TGT​GAC​TCC​AGC​TTA​TCT​CTT​GG
NOX1	TTG​TTT​GGT​TAG​GGC​TGA​ATG​T	GCC​AAT​GTT​GAC​CCA​AGG​ATT​TT
NOX2	TTG​TTT​GGT​TAG​GGC​TGA​ATG​T	GCC​AAT​GTT​GAC​CCA​AGG​ATT​TT
NOX4	CTT​GGT​GAA​TGC​CCT​CAA​CT	TTC​TGG​GAT​CCT​CAT​TCT​GG
GAPDH	GGT​TGT​CTC​CTG​CGA​CTT​CA	GGT​GGT​CCA​GGG​TTT​CTT​ACT​C

### Statistical analysis

Categorical data are shown as the number (%) in the group, and all findings are presented as the mean ± SD. One-way analysis of variance was used to compare differences between multiple groups, and the Student-Newman‒Keuls *post hoc* test was used to evaluate whether the differences were statistically significant. The survival rates of multiple groups were estimated using Kaplan‒Meier survival curves and the log-rank test. Repeated measures MANOVA was conducted on Day 5 for the three groups to assess whether Nrf2 activation reduced weight changes in septic mice. Bonferroni *post hoc* comparisons were used to identify significant differences. A *p*-value of 0.05 was used to determine statistical significance for all two-tailed tests. The figures were created, and statistical analysis was performed using the GraphPad Prism 9.0 program (United States).

## Results

### ATT enhanced liver dysfunction and survival in LPS-induced septic mice

**FIGURE 1:**
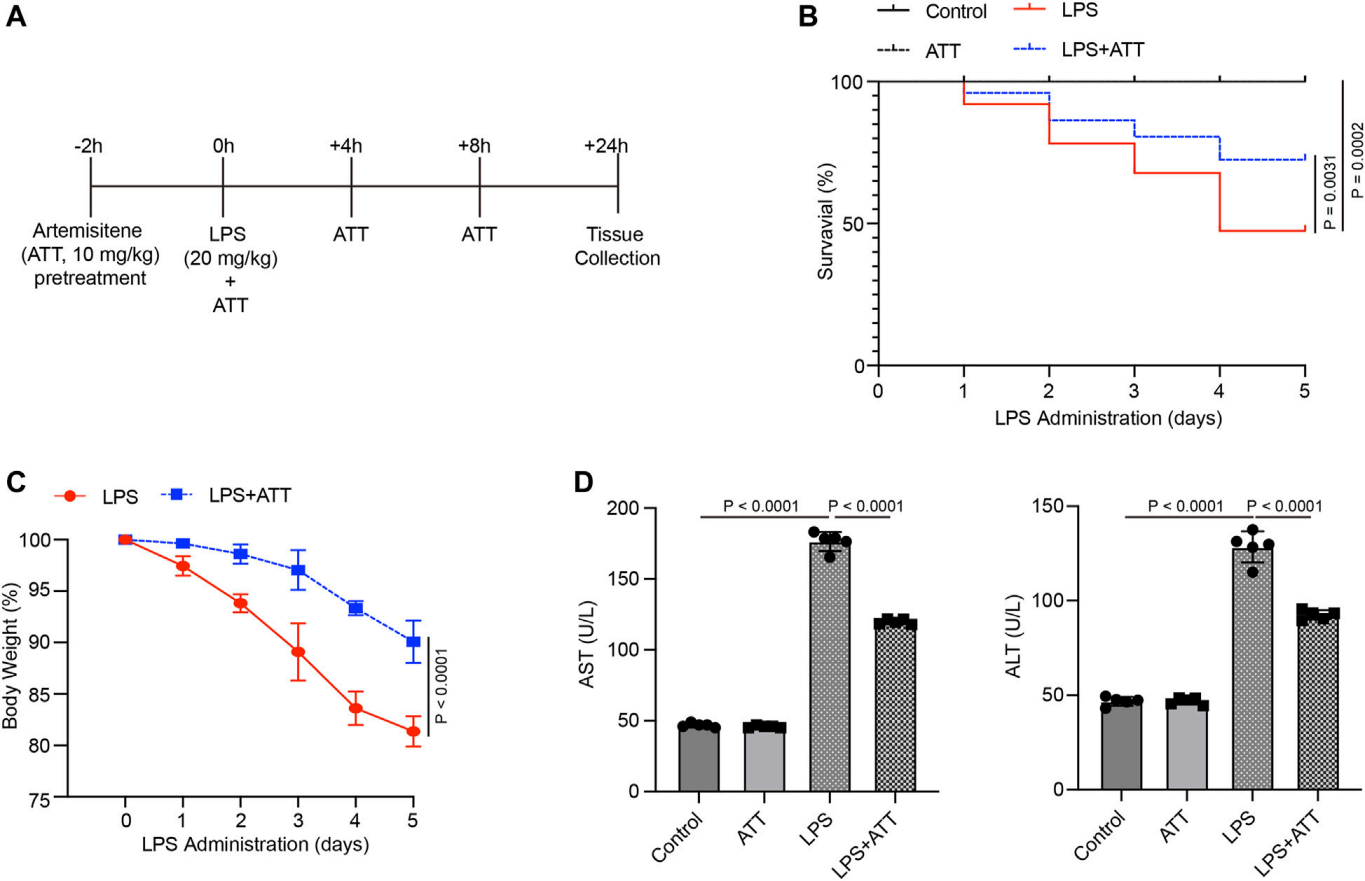
Treatment with Artemisitene improved the survival rate and liver dysfunction in LPS-induced septic mice. **(A)** We treated C57BL/6 with Artemisitene (ATT, 10 mg/kg) 2 h before to the LPS injection, and the injected with LPS (20 mg/kg) intraperitoneally to create a sepsis model for 24 h; **(B)** Survival rate of the control group, ATT, LPS, and LPS + ATT group (*n* = 10); **(C)** The weight changes of mice 5 days after LPS; **(D)** The levels of AST and ALT in each group.

We administered ATT (10 mg/kg) to mice 2 h before LPS injection to determine how it affected LPS-induced liver damage (Figure 1A). According to the findings, LPS-induced mice had a 5-day survival rate of approximately 55%, whereas mice that were given LPS plus ATT had a survival rate of approximately 85% (Figure 1B). The mice’s body weight decreased by 20 percent in the LPS group and by 10 percent in the LPS + ATT group (Figure 1C). The next step was to measure the AST and ALT levels to evaluate liver function. The levels of AST and ALT were considerably higher in the LPS group than in the control group, although the administration of ATT reduced liver dysfunction caused by LPS (Figure 1D).

### ATT reduces oxidative stress in LPS-induced septic animals by activating Nrf2

**FIGURE 2:**
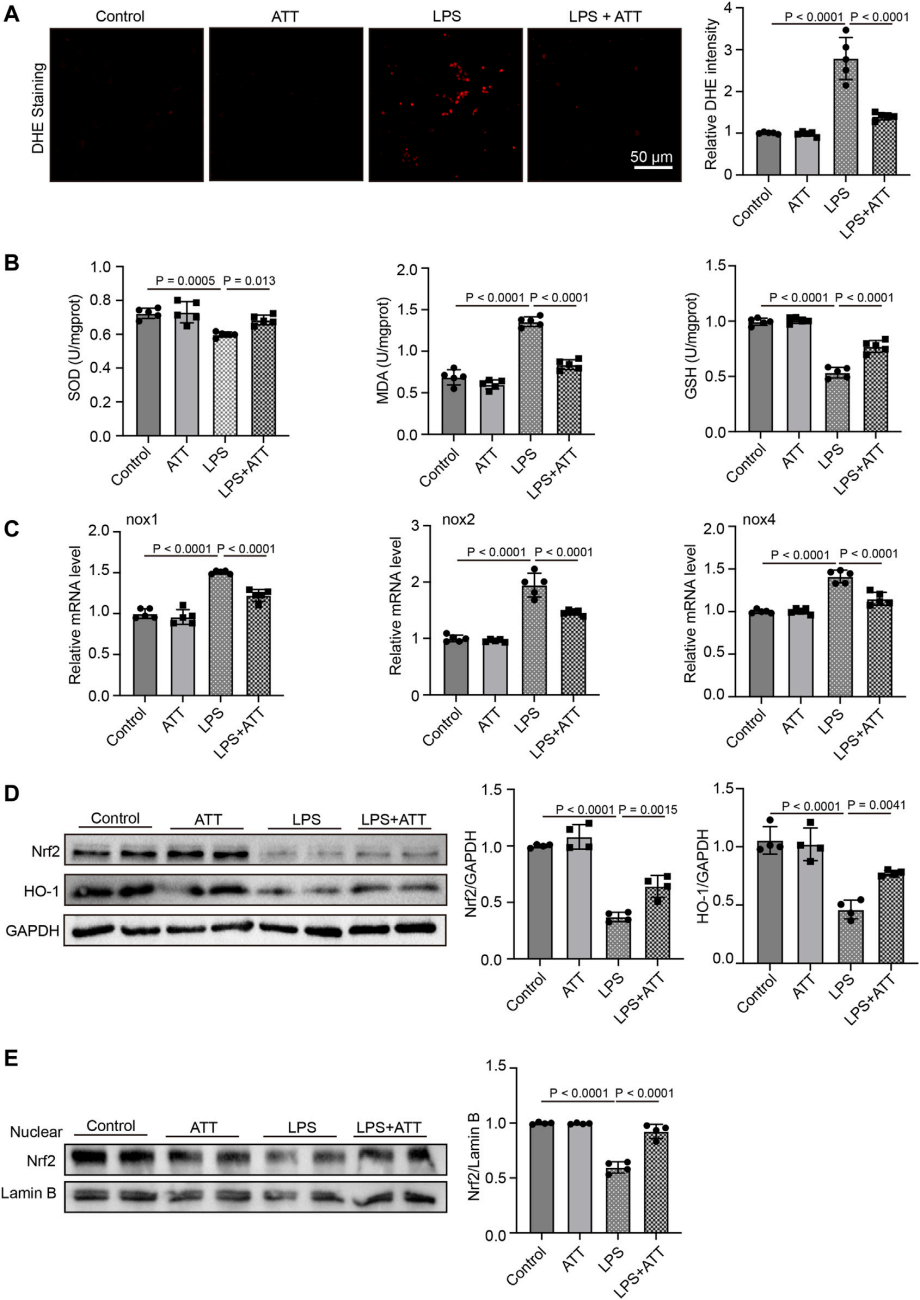
ATT reduces oxidative damage in LPS-induced septic mice. **(A)** DHE staining of representative liver sections (scale bar: 50 μm, *n* = 5); **(B)** The levels of SOD (left), MDA (middle), and GSH in each group (*n* = 5 per group); **(C)** qPCR analyses of nox1, nox2, and nox4 in each group (*n* = 5); **(D)** Western blot analyses of the expression of Nrf2 and HO-1 protein in liver sections (*n* = 4); **(E)** Western blot analyses of the expression of nuclear Nrf2 in liver sections (*n* = 4).

Compared to the control group, DHE staining revealed that the level of DHE in the LPS group was increased, and compared to the LPS group, treatment with ATT reduced the level of DHE (Figure 2A). The levels of SOD, GSH, and MDA in mice treated with LPS were then measured. SOD and GSH levels were both lower in the LPS group than in the control group, whereas MDA levels were higher. Compared to the LPS group, treatment with ATT dramatically increased the levels of SOD and GSH and decreased the level of MDA (Figure 2B). The levels of nox1, nox2 and nox4 mRNA were elevated in the LPS group, as shown in Figure 2C, and ATT could lower these levels. To determine how ATT affects Nrf2 and its downstream protein HO-1, Western blot analysis was used. The results revealed that LPS decreased the expression of Nrf2 and HO-1 and that ATT could stimulate the expression of Nrf2 and HO-1 in comparison to the LPS group (Figure 2D). We next detected the expression of nuclear Nrf2, the result showed that in Figure 2E, LPS decreased the expression of nuclear Nrf2, and ATT treatment increased the expression of nuclear Nrf2.

### ATT reduces liver inflammatory responses in mice with LPS-Induced sepsis

**FIGURE 3:**
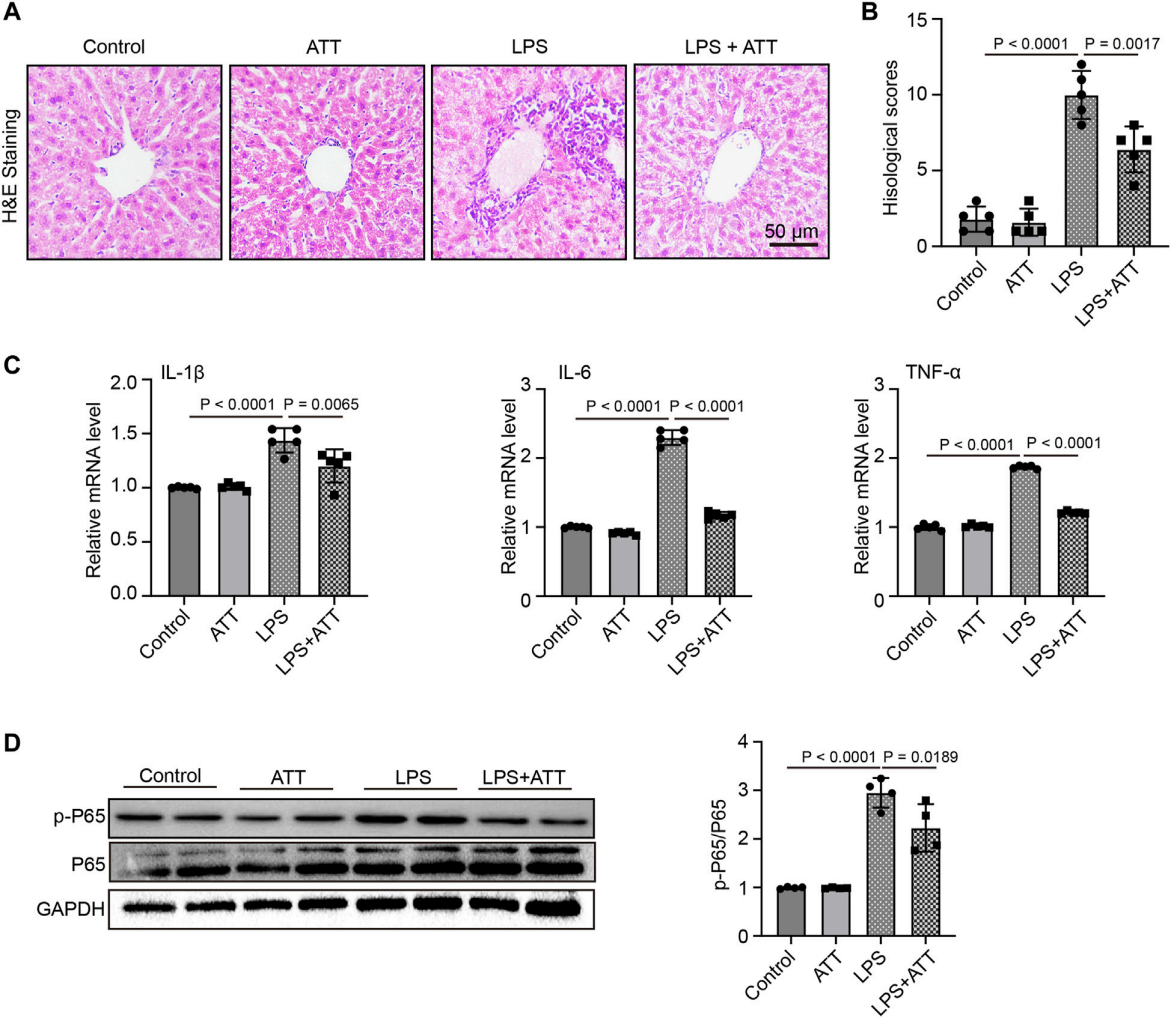
ATT reduces LPS-induced Liver inflammatory injury. **(A)** H&E staining of representative liver sections (scale bar: 50 μm, *n* = 5); **(B)** Histological score (*n* = 5); **(C)** qPCR analyses of IL-1β, IL-6, and TNF-α in each group (*n* = 5); **(D)** Western blot analyses of the expression of p-P65 and P65 protein in liver sections (*n* = 4).

Inflammatory responses are another major cause of LPS-induced harm. LPS significantly increased liver congestion, inflammatory cell infiltration, necrosis, and degeneration, according to H&E staining. ATT might reduce liver inflammation induced by LPS (Figure 3A). In LPS-induced animals, the histological scores revealed significant damage, although ATT reduced this damage compared to the LPS group (Figure 3B). The levels of IL-1β, IL-6, and TNF-α mRNA were then determined. Figure 3C shows that LPS substantially increased the levels of IL-1β, IL-6, and TNF-α mRNA compared to the control group, whereas ATT reduced IL-1β, IL-6, and TNF-α mRNA levels. Similarly, western blotting demonstrated that ATT suppressed the expression of p-P65, whereas LPS enhanced its expression (Figure 3D). According to the findings, the inflammatory reactions of LPS-induced septic mice might be reduced by activating Nrf2.

### ATT reduces ferroptosis in septic mice caused by LPS

**FIGURE 4:**
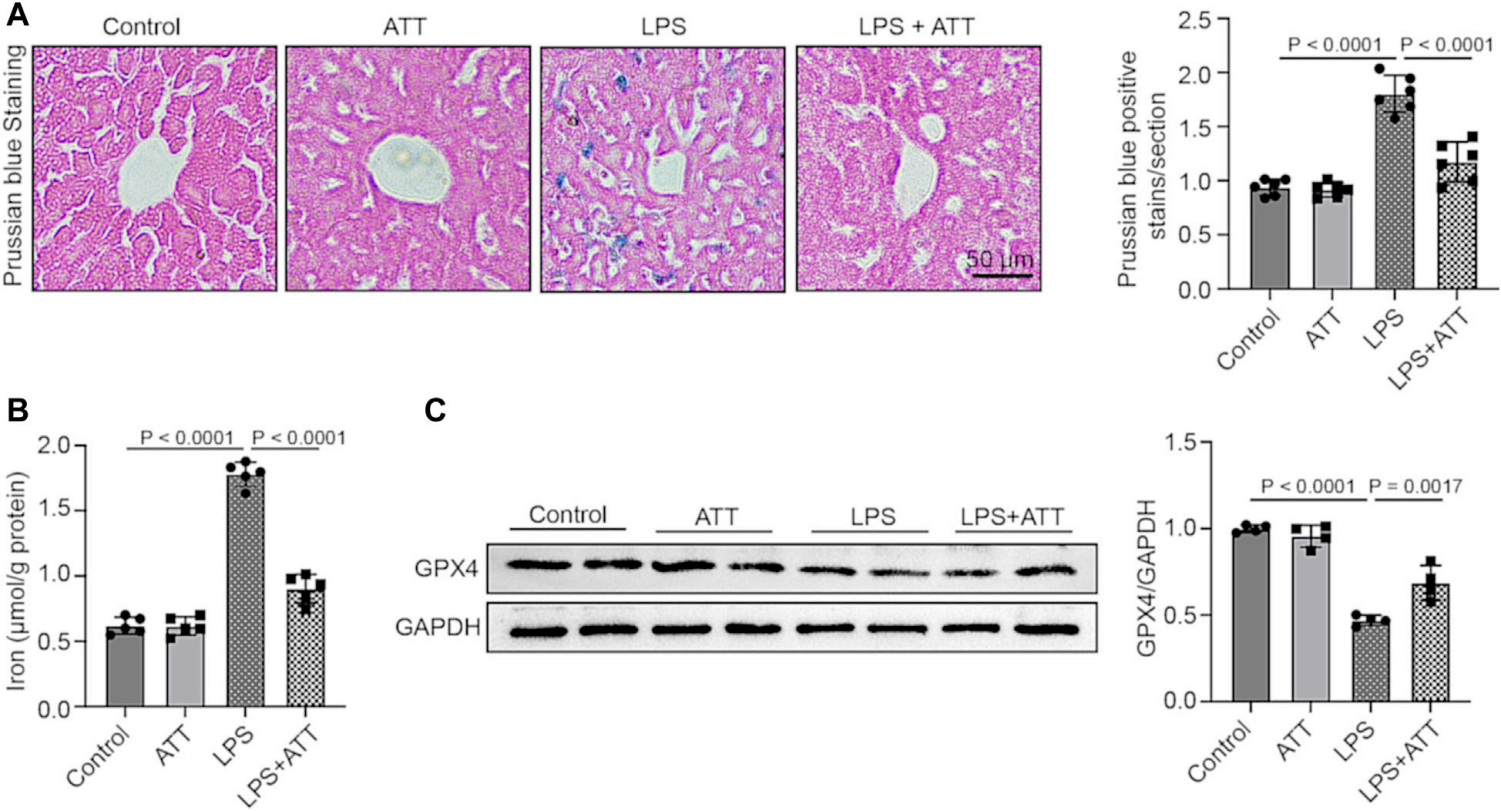
**(A)** Prussian blue staining of representative liver sections (scale bar: 50 μm, *n* = 5); **(B)** The level of serum iron in each group (*n* = 5); **(C)** Western blot analyses of the expression of GPX4 protein in liver sections (*n* = 4).

Prussian blue staining was used to determine the amount of iron deposited in the liver. The results demonstrated that ATT substantially decreased iron deposition in mice treated with LPS and that LPS increased iron deposition in the liver relative to the control group (Figure 4A). The mice in the LPS group had considerably more serum iron in comparison to the control group, but less serum iron was present after ATT administration (Figure 4B). In addition, we measured the protein expression of GPX4. As shown in Figure 4C, the LPS group had lower GPX4 expression levels, but ATT increased GPX4 expression levels in comparison to the LPS group.

### ATT suppressed LPS-induced ferroptosis *in vitro*

**FIGURE 5:**
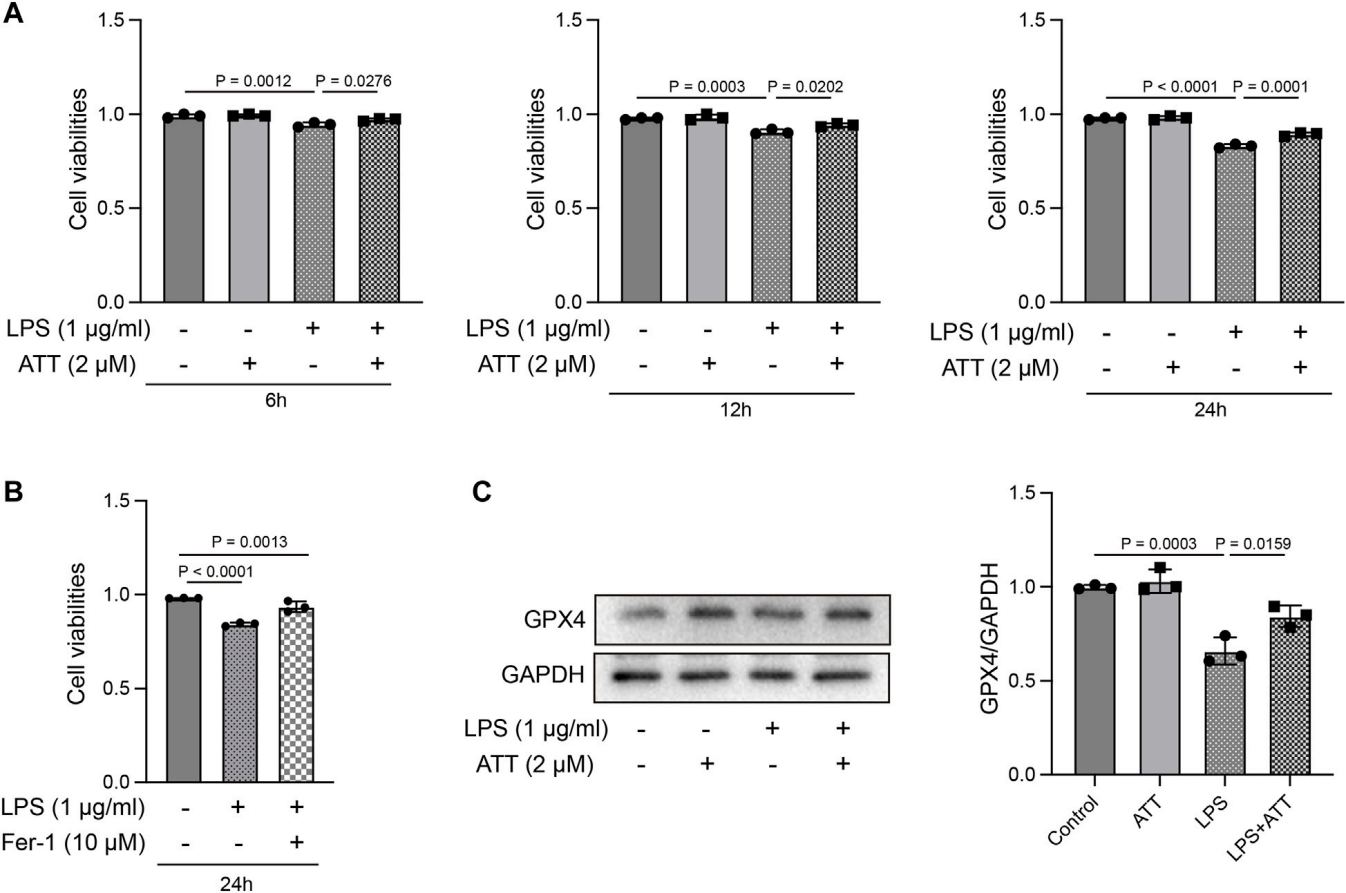
Effect of ATT treatment on cell viability in LPS-treated L-02 cells. **(A, B)** MTT assay showed ATT inhibited L-02 cell death caused by LPS at 6, 12, and 24 h (*n* = 3); **(C)** Western blot analyses of the expression of GPX4 protein in L-02 cells (*n* = 3).

We examined the ways through which ATT inhibits ferroptosis *in vitro*. At 6, 12, and 24 h, the MTT assay revealed that ATT (2 μM) prevented LPS (1 μg/mL)-induced L-02 cell death (Figure 5A). We treated cells with LPS and Fer-1 (10 μM) to further demonstrate that LPS causes ferroptosis. The findings revealed that Fer-1 decreased LPS-induced cell death, demonstrating that LPS induced ferroptosis (Figure 5B). ATT rescued the LPS-induced decrease in GPX4 protein expression in L-02 cells, as shown by Western blotting (Figure 5C).

### Activation of Nrf2 by ATT reduces inflammation and oxidative stress responses *in vitro*

**FIGURE 6:**
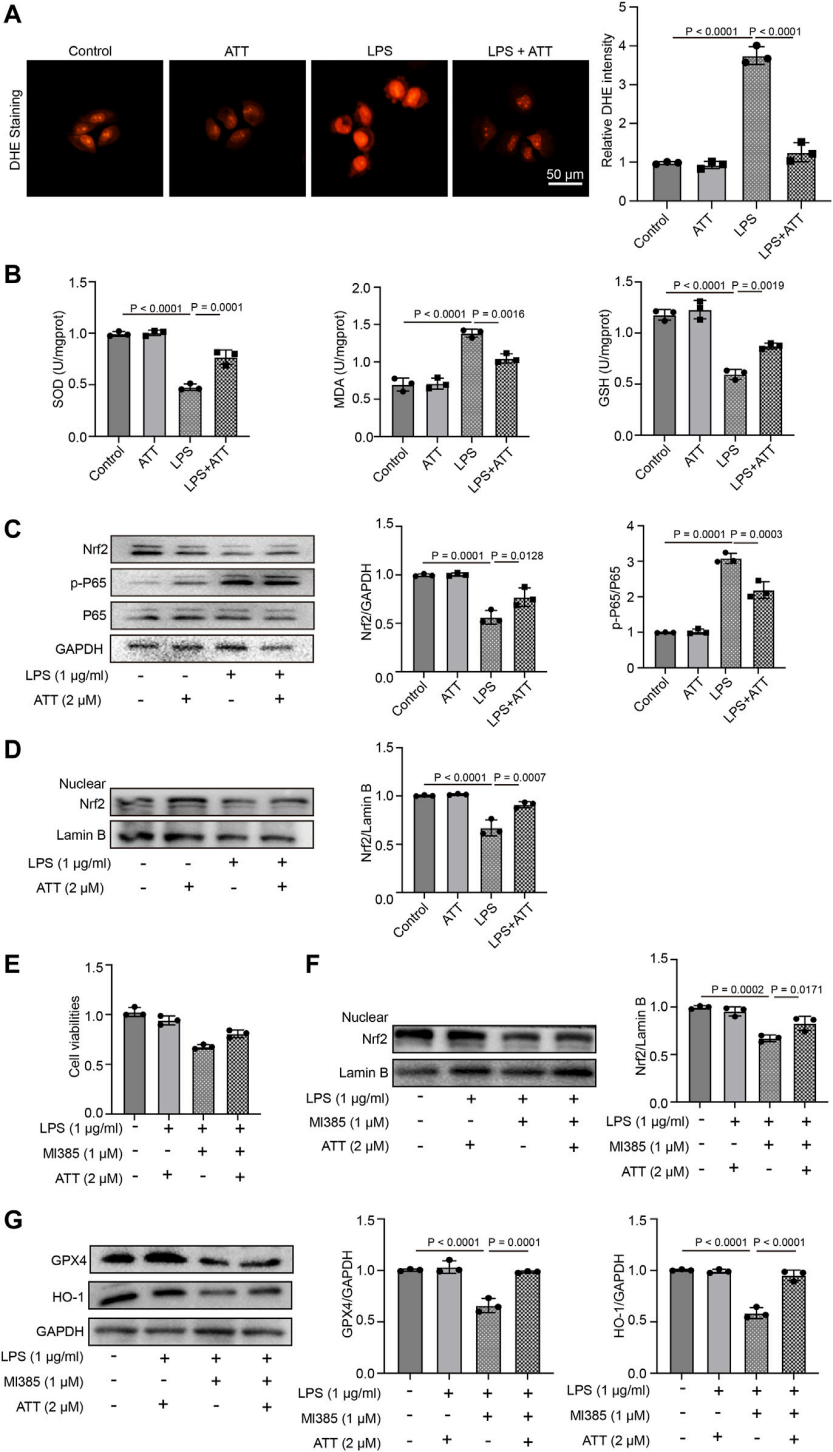
ATT increase the expression of Nrf2, prevent the oxidative damage, and inflammation in LPS-treated L-02 cells. **(A)** DHE staining of L-02 cells (*n* = 3); **(B)** The levels of SOD (left), MDA (middle), and GSH in each group (*n* = 3 per group); **(C)** Western blot analyses of the expression of Nrf2, P65, and p-P65 protein in L-02 cells (*n* = 3); **(D)** Western blot analyses of the expression of nuclear Nrf2 in L-02 cells (*n* = 3); **(E)** The L-02 cells were treated with ATT, Nrf2 inhibitor - Ml385, and LPS, MTT assay detected the cell viability of L-02 cells (*n* = 3); **(F-G)** Western blot analyses of the expression of nuclear Nrf2, GPX4 and HO-1 (*n* = 3).

We first treated L-02 cells with ATT for 8 h, followed by 24 h of treatment with LPS (1 μg/mL). DHE staining demonstrated that ATT reduced the amount of DHE in cells that were subjected to LPS (Figure 6A). We measured the levels of SOD, GSH, and MDA. As shown in Figure 6B, LPS decreased in the levels of SOD and GSH and increased the level of MDA. ATT could counteract this effect by increasing the levels of SOD and GSH and decreasing the level of MDA. Western blotting demonstrated that LPS increased p-P65 expression, while LPS reduced Nrf2 expression, and ATT had the opposite effect (Figure 6C). We detected the expression of nuclear Nrf2, the result showed in Figure 6D, ATT treatment increased LPS-induced reduction of nuclear Nrf2 level. To further confirmed the effects of ATT, we treated the cells with Nrf2 inhibitor—Ml385. The result showed treatment with LPS and Ml385 could significantly reduce the cell viability compared with control group, and in ATT + Ml385+ LPS group, the cell viability was increased compared with LPS + MI385 group (Figure 6E). We next detected the expression of nuclear Nrf2, GPX4 and HO-1, the result showed in Figures 6F,G, the treatment of LPS and Ml385 could decrease the expression of nuclear Nrf2, GPX4 and HO-1 compared with control group, and in ATT + Ml385+ LPS group, the expression of nuclear Nrf2, GPX4 and HO-1 were increased.

## Discussion

**FIGURE 7:**
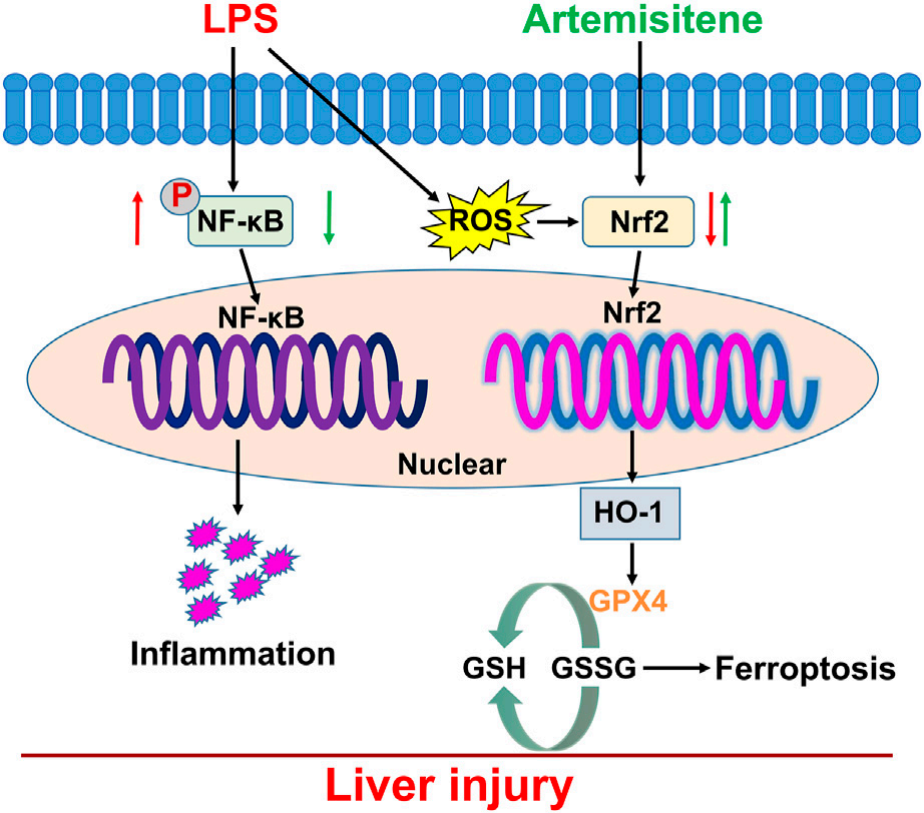
Working model for ATT in the regulation of LPS-induced sepsis model. LPS treatment induced oxidative stress, inflammatory responses, and ferroptosis which resulted in liver injury and dysfunction through downregulation of Nrf2 and NF-κB. ATT could rescue oxidative stress and inflammatory responses, and ferroptosis through increasing Nrf2, HO-1 and GPX4, and decreasing NF-κB. ATT could be used as an agent to protect against LPS-induced septic liver injury and dysfunction.

Sepsis is a severe infection-related response that has a high fatality rate and requires immediate measures to improve outcomes. The goal of the global campaign Surviving Sepsis is to improve sepsis patient outcomes, but the disease pathophysiology is still largely unknown (Purcarea and Sovaila, 2020). The largest organ in the human body is the liver, which is crucial for maintaining immune and metabolic balance (Strnad P et al., 2017). During sepsis, infections, poisons, or inflammatory mediators damage the liver. Liver failure follows liver damage, which develops from active hepatocellular dysfunction in the injury (Kim and Choi, 2020). Ferroptosis is caused by the elevation of iron levels and excessive accumulation of lipid ROS (Wei S et al., 2020). GPX4 is a lipid repair enzyme that can GSH to oxidized glutathione disulfide (GSSG), eliminate lipid peroxides, and protect cell membrane against peroxidation of polyunsaturated fatty acids. Several studies have demonstrated that the GPX4 is the key upstream regulator of ferroptosis. It has been reported that GPX4 activity decreases can lead to ferroptosis (Yang WS et al., 2014; Seibt TM et al., 2019). The liver is the major extraerythrocyte storage organ for iron (Ishizaka N et al., 2005). Recent studies have found that hepatic iron overload always leads to oxidative stress, which has been found to be involved in the progression of liver disease (Jiang H et al., 2022). However, whether iron disorder is involved in acute liver disease and the further molecular mechanisms remain unclear. Thus, it is essential to continue researching the mechanism of LPS-induced hepatotoxicity and create sepsis treatments. In the current investigation, we demonstrated the mechanism of LPS-induced hepatotoxicity and used ATT to protect against LPS-induced liver damage. We discovered that ATT significantly reduced the liver damage caused by LPS by reducing ROS and inflammatory responses and by activating the Nrf2/GPX4 and NF-κB pathways (Figure 7).

By regulating genes involved in the response to oxidative stress and drug detoxification, Nrf2 modulates the cellular defense against toxic and oxidative assaults (He et al., 2020). Cells become resistant to chemical carcinogens and inflammatory threats when Nrf2 is activated. Nrf2 participates in a wide range of physiological activities, including inflammation and metabolism, in addition to antioxidant responses (Ahmed et al., 2017). Several cell defense genes, including NF-κB, have AREs in the promoter region that Nrf2 can bind to and initiate their transcription. The antioxidant gene Nrf2 and its downstream protein HO-1, which have anti-inflammatory properties, protect cells from a variety of types of damage. According to many studies, increasing HO-1 expression through Nrf2 activation inhibits NF-κB signaling, which in turn reduces inflammatory responses in a rat liver transplantation model (Ren et al., 2020). In our study, we discovered that LPS could cause inflammation and increase NF-κB expression by inhibiting the expression of Nrf2 and HO-1. In LPS-induced septic mice, treatment with ATT could increase the expression of Nrf2 and HO-1 to reduce inflammation and NF-κB expression (Figures 2, 3).

The vulnerability of cells to ferroptosis is primarily controlled by ROS and GPX4, and ROS play a significant role in LPS-induced liver damage (Yang et al., 2022). In the presence of GSH, GPX4 may convert harmful lipid ROS to harmless lipid alcohols, avoiding ferroptosis. The ratio of GSH/GSSG is a good biomarker of redox homeostasis in the liver because GSH may be transformed into GSSG in the presence of harmful stimuli. GSH depletion results in the inactivation of GPX4, an increase in cellular lipid peroxidation, and ferroptosis (Li, et al., 2022). DHE staining showed that LPS could increase ROS levels. The GPX4 protein was then inactivated, and ferroptosis occurred in LPS-treated mice as a result of the reductions in SOD and GSH being and the increase in MDA (Figures 2, 4). ATT is a byproduct of the antimalarial drug artemisinin and is found in the plant known as sweet wormwood (Hsu, 2016). ATT is an antioxidant and anticancer agent that activates Nrf2. ATT stimulates Nrf2 by decreasing Nrf2 ubiquitination and increasing its stability (Chen, et al., 2016). According to several studies, Nrf2 plays a crucial role in reducing peroxidation and ferroptosis (Dodson et al., 2019). In the current study, we pretreated the mice and cells with ATT, because sepsis can trigger acute-on-chronic liver failure - a syndrome with high short-term mortality (Strnad P et al., 2017). ATT with the dose of 10 mg/kg can effectively activate the Nrf2-dependent antioxidant response *in vivo*. No obvious toxicity was observed in lung, kidney, and liver tissues of mice (Chen W et al., 2016). In our study, we discovered that ATT could reduce ROS and MDA levels while increasing GSH, SOD, and GPX4 protein expression levels. It was hypothesized that in LPS-induced septic mice, ATT increased the expression of Nrf2/HO-1 and that increasing Nrf2 reduced ROS and ferroptosis.

Our findings offer a fresh understanding of how ferroptosis contributes to the onset and progression of LPS-induced liver damage. Moreover, we showed that ATT inhibited ROS and inflammation by activating Nrf2/HO-1/GPX4 and downregulating NF-κB, which protected against liver damage caused by ferroptosis. Our work was limited in that we only examined how ATT affected the Nrf2/HO-1/GPX4 signaling pathway; however, other signaling pathways may also impact ferroptosis; therefore, it is still unclear how much liver damage ATT prevents by preventing ferroptosis.

## Data Availability

The original contributions presented in the study are included in the article/Supplementary Materials, further inquiries can be directed to the corresponding authors.
