# Supplementary material for: Artemisitene Alters LPS-Induced Oxidative stress, inflammation and Ferroptosis in Liver Through Nrf2/HO-1 and NF-kB Pathway
Source: Front Pharmacol. 2023 Apr 25;14:1177542. doi: 10.3389/fphar.2023.1177542 (PMC10167592; doi:10.3389/fphar.2023.1177542)
Supplement: Supplementary file 1 [file DataSheet1.PDF]

## Figure1B the number of death

| | 0day | 1 day | 2day | 3day | 4day | 5 day |
|---|---|---|---|---|---|---|
| Control | 0 | 0 | 0 | 0 | 0 | 0 |
| ATT | 0 | 0 | 0 | 0 | 0 | 0 |
| LPS | 0 | 2 | 3 | 2 | 3 | 0 |
| LPS+ATT | 0 | 1 | 2 | 1 | 1 | 0 |

## Figure1C

| Body weight | 0day | % | 1 day | % | 2day | % | 3day | % | 4day | % | 5 day | % |
|---|---|---|---|---|---|---|---|---|---|---|---|---|
| LPS 1 | 2400 | 100 | 2324 | 96.83 | 2247.000 | 93.63 | 2180 | 90.83 | 1980 | 82.50 | 1975 | 82.29 |
| LPS 2 | 2340 | 100 | 2305 | 98.50 | 2219.000 | 94.83 | 2120 | 90.60 | 1988 | 84.96 | 1939 | 82.86 |
| LPS 3 | 2375 | 100 | 2338 | 98.44 | 2230.000 | 93.89 | 2170 | 91.37 | 1956 | 82.36 | 1876 | 78.99 |
| LPS 4 | 2330 | 100 | 2259 | 96.95 | 2197.000 | 94.29 | 1975 | 84.76 | 1921 | 82.45 | 1897 | 81.42 |
| LPS 5 | 2270 | 100 | 2192 | 96.56 | 2099.000 | 92.47 | 1995 | 87.89 | 1947 | 85.77 | 1845 | 81.28 |
| LPS 6 | 2397 | 100 | 2310 | 96.37 | 2210.000 | 92.20 | 2140 | 89.28 | 2000 | 83.44 | 1890 | 78.85 |
| LPS+ATT 1 | 2356 | 100 | 2340 | 99.32 | 2290.000 | 97.20 | 2231 | 94.69 | 2198 | 93.29 | 2054 | 87.18 |
| LPS+ATT 2 | 2404 | 100 | 2402 | 99.92 | 2397.000 | 99.71 | 2378 | 98.92 | 2249 | 93.55 | 2156 | 89.68 |
| LPS+ATT 3 | 2420 | 100 | 2410 | 99.59 | 2399.000 | 99.13 | 2305 | 95.25 | 2237 | 92.44 | 2167 | 89.55 |
| LPS+ATT 4 | 2340 | 100 | 2333 | 99.70 | 2310.000 | 98.72 | 2295 | 98.08 | 2178 | 93.08 | 2138 | 91.37 |
| LPS+ATT 5 | 2294 | 100 | 2285 | 99.61 | 2256.000 | 98.34 | 2256 | 98.34 | 2164 | 94.33 | 2125 | 92.63 |

## Figure 1D AST

| **AST** | Control | ATT | LPS | LPS+ATT |
|---|---|---|---|---|
| 1 | 47 | 45 | 178.91 | 121.5 |
| 2 | 49 | 47 | 183.24 | 119.47 |
| 3 | 45 | 46 | 176.48 | 118.37 |
| 4 | 46 | 46 | 165.39 | 121.75 |
| 5 | 47 | 45 | 178.57 | 117.98 |

## ALT

| ALT | Control | ATT | LPS | LPS+ATT |
|---|---|---|---|---|
| 1 | 43.12 | 44.38 | 129.77 | 93.29 |
| 2 | 49.45 | 48.57 | 128.36 | 93.47 |
| 3 | 47.63 | 48.43 | 131.37 | 95.57 |
| 4 | 47.34 | 47.39 | 137.58 | 90.48 |
| 5 | 46.46 | 45.38 | 115.09 | 89.42 |

Figure 2A

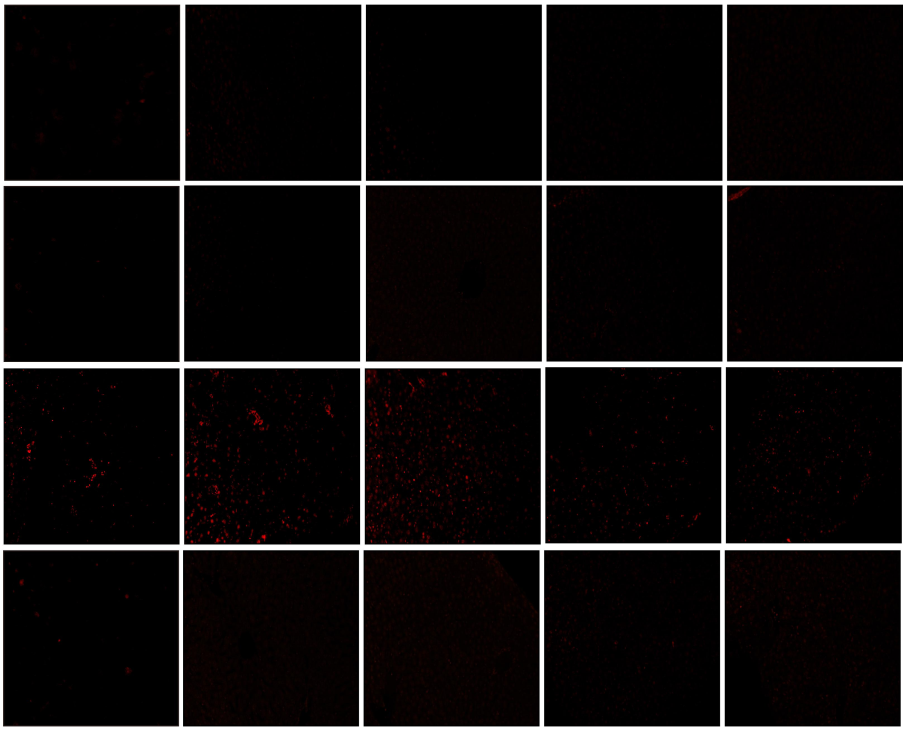


| DHE | Control | ATT | LPS | LPS+ATT |
|---|---|---|---|---|
| 1 | 1.013 | 0.987 | 2.513 | 1.513 |
| 2 | 0.974 | 0.899 | 2.145 | 1.432 |
| 3 | 0.996 | 0.979 | 2.769 | 1.276 |
| 4 | 1.013 | 1.014 | 3.467 | 1.401 |
| 5 | 1.034 | 1.045 | 3.057 | 1.398 |

**Figure 2B**

| MDA | Control | ATT | LPS | LPS+ATT |
|---|---|---|---|---|
| 1 | 0.549 | 0.643 | 1.321 | 0.796 |
| 2 | 0.678 | 0.576 | 1.369 | 0.843 |
| 3 | 0.713 | 0.515 | 1.432 | 0.912 |
| 4 | 0.695 | 0.611 | 1.278 | 0.874 |
| 5 | 0.801 | 0.653 | 1.379 | 0.766 |

| GSH | Control | ATT | LPS | LPS+ATT |
|---|---|---|---|---|
| 1 | 0.986 | 1.035 | 0.476 | 0.697 |
| 2 | 0.945 | 0.969 | 0.545 | 0.781 |
| 3 | 1.031 | 1.032 | 0.573 | 0.835 |
| 4 | 0.996 | 0.998 | 0.492 | 0.798 |
| 5 | 1.012 | 1 | 0.583 | 0.745 |

| SOD | Control | ATT | LPS | LPS+ATT |
|---|---|---|---|---|
| 1 | 0.756 | 0.801 | 0.598 | 0.673 |
| 2 | 0.743 | 0.787 | 0.575 | 0.703 |

| | | | | |
|---|---|---|---|---|
| 3 | 0.732 | 0.649 | 0.617 | 0.723 |
| 4 | 0.678 | 0.702 | 0.604 | 0.679 |
| 5 | 0.711 | 0.714 | 0.598 | 0.647 |

## Figure 2C

| NOX1 | Control | Relative | ATT | Relative | LPS | Relative | LPS+ATT | Relative |
|---|---|---|---|---|---|---|---|---|
| 1 | 12.997 | 0.948 | 13.336 | 0.972 | 20.889 | 1.523 | 16.788 | 1.224 |
| 2 | 14.580 | 1.063 | 13.070 | 0.953 | 20.335 | 1.483 | 17.716 | 1.292 |
| 3 | 14.580 | 1.063 | 15.128 | 1.103 | 20.720 | 1.511 | 17.389 | 1.268 |
| 4 | 13.336 | 0.972 | 12.107 | 0.883 | 20.889 | 1.523 | 16.788 | 1.224 |
| 5 | 13.070 | 0.953 | 12.107 | 0.883 | 20.335 | 1.483 | 15.128 | 1.103 |
| NOX2 | | | | | | | | |
| | Control | Relative | ATT | Relative | LPS | Relative | LPS+ATT | Relative |
| 1 | 14.99292278 | 0.947120833 | 15.54245853 | 0.982 | 31.67825317 | 2.001 | 22.84858894 | 1.443 |
| 2 | 17.26961136 | 1.090941968 | 14.88915253 | 0.941 | 31.76664352 | 2.007 | 22.01982689 | 1.391 |
| 3 | 15.78096867 | 0.996902632 | 14.7366066 | 0.931 | 26.58437347 | 1.679 | 23.85477638 | 1.507 |
| 4 | 16.08510971 | 1.016115585 | 15.6167345 | 0.987 | 28.71092796 | 1.814 | 23.55155945 | 1.488 |
| 5 | 15.01830006 | 0.948723945 | 15.13666153 | 0.956 | 35.30083847 | 2.230 | 23.38820648 | 1.477 |
| NOX4 | | | | | | | | |
| | Control | Relative | ATT | Relative | LPS | Relative | LPS+ATT | Relative |
| 1 | 23.92093849 | 1.003816135 | 23.61239243 | 0.990868335 | 35.36696243 | 1.484136065 | 26.25689507 | 1.101842009 |
| 2 | 23.40601921 | 0.982208108 | 22.83978653 | 0.95844677 | 34.84534836 | 1.462247099 | 29.85573387 | 1.25286336 |
| 3 | 23.78839493 | 0.998254088 | 24.70027542 | 1.03652016 | 31.67825317 | 1.329343398 | 26.58437347 | 1.115584283 |
| 4 | 23.38820648 | 0.981460616 | 24.66200447 | 1.034914162 | 31.76664352 | 1.333052603 | 28.71092796 | 1.204822827 |
| 5 | 24.63288689 | 1.033692274 | 23.85477638 | 1.001039714 | 34.47117233 | 1.446545209 | 25.58511734 | 1.073651588 |

## Figure 2D WB

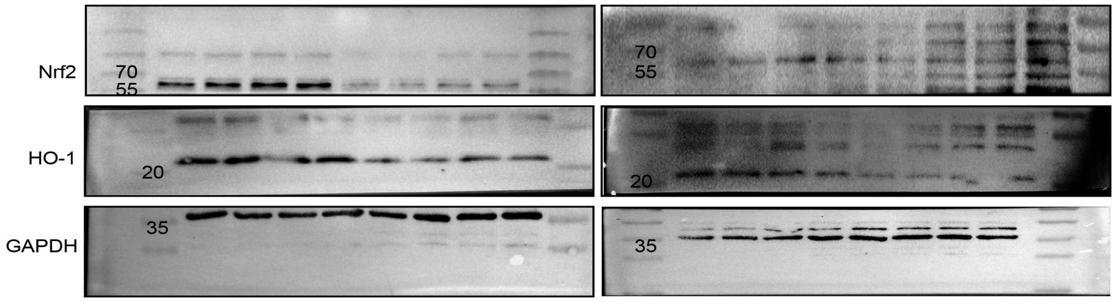

| Nrf2 | Control | ATT | LPS | LPS+ATT |
|---|---|---|---|---|
| 1 | 1.011 | 1.201 | 0.347 | 0.507 |
| 2 | 0.998 | 1.145 | 0.401 | 0.642 |
| 3 | 0.979 | 0.986 | 0.325 | 0.731 |
| 4 | 1.013 | 0.992 | 0.413 | 0.688 |

| HO-1 | Control | ATT | LPS | LPS+ATT |
|---|---|---|---|---|
| 1 | 1.013 | 0.874 | 0.365 | 0.783 |
| 2 | 0.978 | 1.21 | 0.432 | 0.749 |
| 3 | 1.231 | 1.015 | 0.545 | 0.804 |
| 4 | 1.001 | 0.989 | 0.506 | 0.763 |

Figure 2E

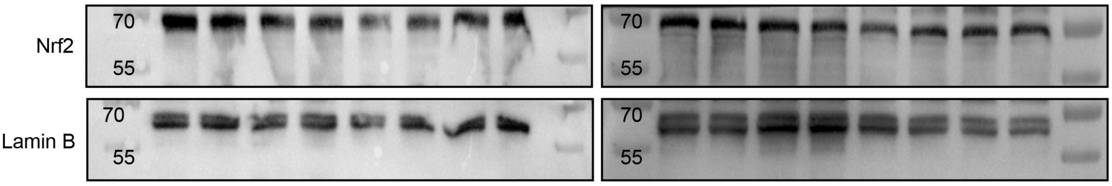


| Nu NRF2 | Control | ATT | LPS | LPS+ATT |
|---|---|---|---|---|
| 1 | 1.01 | 0.992 | 0.644 | 0.934 |
| 2 | 1.001 | 0.989 | 0.632 | 0.956 |
| 3 | 0.987 | 1.003 | 0.579 | 0.831 |
| 4 | 0.995 | 0.997 | 0.531 | 0.977 |

Figure 3A

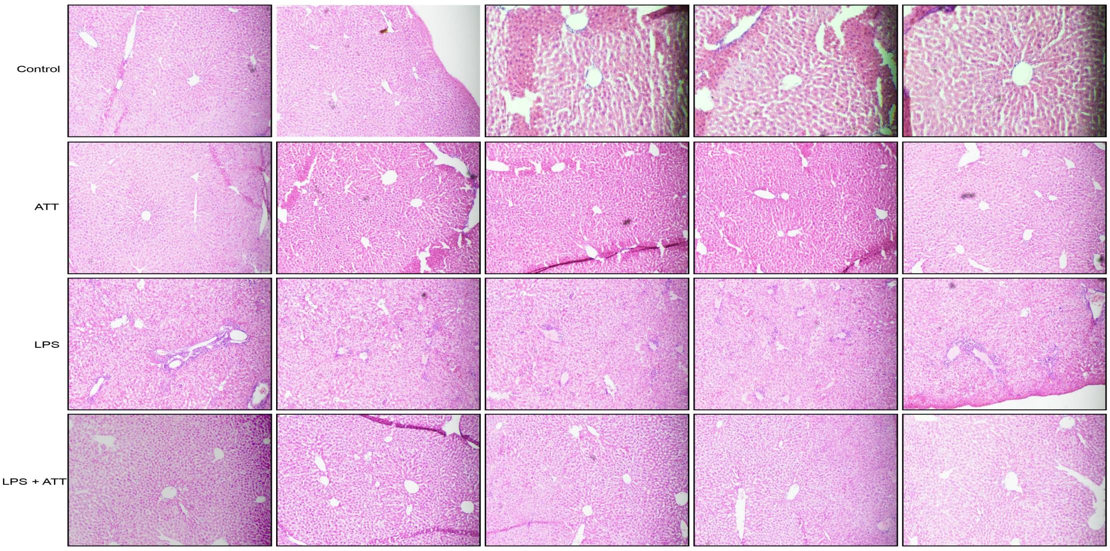


Figure 3B

| Scores | Control | ATT | LPS | LPS+ATT |
|---|---|---|---|---|
| 1 | 2 | 1 | 12 | 8 |
| 2 | 3 | 2 | 8 | 7 |
| 3 | 1 | 3 | 10 | 6 |
| 4 | 2 | 1 | 11 | 7 |
| 5 | 1 | 1 | 9 | 4 |

## Figure 3C

| IL-1β | Control | Relative | ATT | Relative | LPS | Relative | LPS+ATT | Relative |
|---|---|---|---|---|---|---|---|---|
| 1 | 27.40 | 1.010 | 27.17166667 | 1.001314367 | 41.77601242 | 1.539505175 | 25.30083847 | 0.932371701 |
| 2 | 27.25 | 1.004 | 26.24 | 0.966981132 | 38.58538055 | 1.421925875 | 35.04878616 | 1.291597367 |
| 3 | 26.80 | 0.988 | 28.43 | 1.047685731 | 34.28294373 | 1.26337499 | 35.29603195 | 1.300708725 |
| 4 | 27.16 | 1.001 | 27.67 | 1.019678656 | 41.77601242 | 1.539505175 | 33.82939911 | 1.246661229 |
| 5 | 27.07 | 0.998 | 27.45 | 1.011571344 | 38.58538055 | 1.421925875 | 33.43413162 | 1.232095063 |

| IL-6 | Control | Relative | ATT | Relative | LPS | Relative | LPS+ATT | Relative |
|---|---|---|---|---|---|---|---|---|
| 1 | 18.80534363 | 0.9856 | 17.71584511 | 0.928357444 | 45.83042145 | 2.401636087 | 21.18 | 1.110089698 |
| 2 | 19.17533684 | 1.0050 | 17.3885231 | 0.9112049 | 45.86901093 | 2.403658278 | 23.54 | 1.233416384 |
| 3 | 19.45610809 | 1.0197 | 16.78759766 | 0.879714807 | 43.48210907 | 2.278578267 | 21.75 | 1.139882737 |
| 4 | 18.80534363 | 0.9856 | 17.71584511 | 0.928357444 | 43.17362213 | 2.26241273 | 22.51 | 1.17971367 |
| 5 | 19.17533684 | 1.0050 | 17.3885231 | 0.9112049 | 41.0125618 | 2.149167416 | 22.83 | 1.196383555 |

| TNF-α | Control | Relative | ATT | Relative | LPS | Relative | LPS+ATT | Relative |
|---|---|---|---|---|---|---|---|---|
| 1 | 24.93 | 1.033401726 | 24.31 | 1.007879393 | 44.36 | 1.838931436 | 28.93 | 1.199492649 |
| 2 | 25.31 | 1.049248298 | 24.55 | 1.017800058 | 45.25 | 1.875945861 | 29.98 | 1.243027328 |
| 3 | 23.55 | 0.976561393 | 24.18 | 1.002363028 | 44.73 | 1.854544493 | 28.90 | 1.198145169 |
| 4 | 24.05 | 0.997291394 | 24.01 | 0.995344586 | 45.53 | 1.887846705 | 29.08 | 1.205467347 |
| 5 | 22.78 | 0.944488203 | 25.39 | 1.052674172 | 45.59 | 1.890333691 | 29.21 | 1.211067772 |

Figure 3D

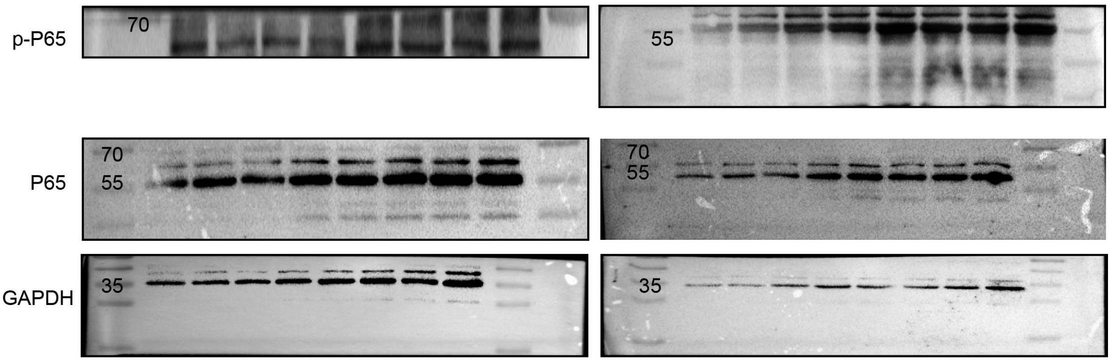


| p-P65/P65 | Control | ATT | LPS | LPS+ATT |
|---|---|---|---|---|
| 1 | 1.001 | 1.002 | 3.245 | 2.478 |
| 2 | 0.987 | 0.984 | 3.078 | 2.796 |
| 3 | 0.965 | 0.98 | 2.946 | 1.873 |
| 4 | 1.013 | 0.976 | 2.531 | 1.765 |

Figure 4A

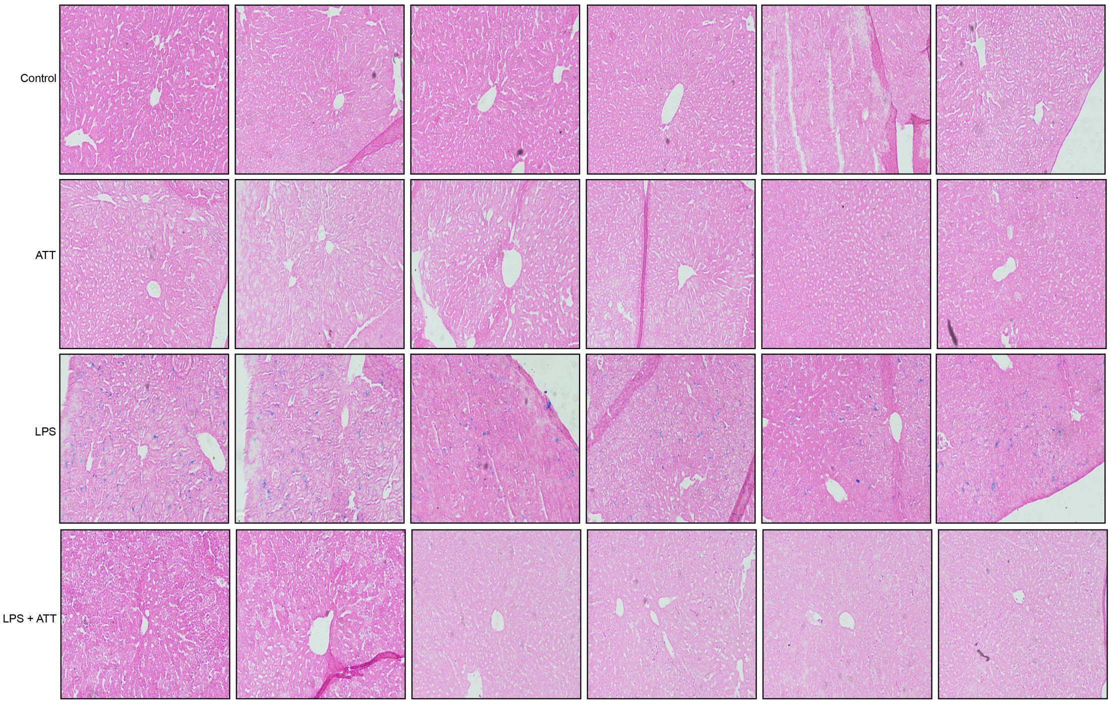


| | Control | ATT | LPS | LPS+ATT |
|---|---|---|---|---|
| 1 | 1.01 | 0.951 | 2.037 | 0.935 |
| 2 | 0.974 | 0.845 | 1.684 | 1.234 |
| 3 | 0.836 | 0.832 | 1.573 | 1.406 |
| 4 | 1.012 | 1.011 | 1.945 | 1.321 |
| 5 | 0.857 | 0.938 | 1.832 | 0.992 |
| 6 | 0.942 | 0.915 | 1.745 | 1.153 |

Figure 4B Iron

| | Control | ATT | LPS | LPS+ATT |
|---|---|---|---|---|
| 1 | 0.672 | 0.674 | 1.877 | 1.013 |
| 2 | 0.577 | 0.596 | 1.764 | 0.978 |
| 3 | 0.601 | 0.699 | 1.635 | 0.842 |
| 4 | 0.549 | 0.523 | 1.798 | 0.936 |
| 5 | 0.701 | 0.599 | 1.832 | 0.745 |

Figure 4C

GPX4

GAPDH

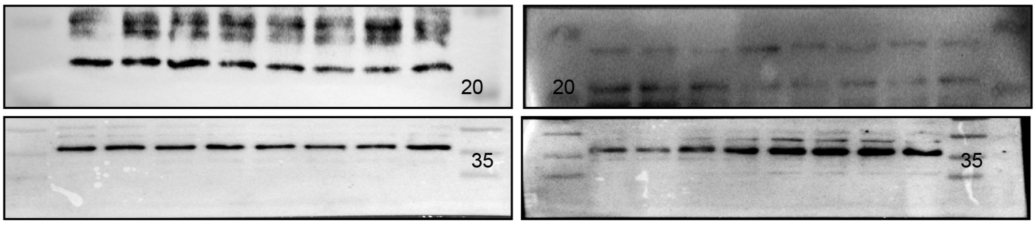


| GPX4 | Control | ATT | LPS | LPS+ATT |
|---|---|---|---|---|
| 1 | 1.021 | 0.872 | 0.431 | 0.645 |
| 2 | 0.986 | 0.945 | 0.502 | 0.812 |
| 3 | 1.013 | 1.011 | 0.467 | 0.576 |
| 4 | 0.974 | 0.998 | 0.478 | 0.712 |

Figure 5A

| 6h | Control | ATT | LPS | LPS+ATT |
|---|---|---|---|---|
| 1 | 1 | 1 | 0.932 | 0.977 |
| 2 | 0.99 | 0.99 | 0.956 | 0.963 |
| 3 | 0.98 | 0.99 | 0.945 | 0.974 |
| 12h | Control | ATT | LPS | LPS+ATT |
| 1 | 0.97 | 0.98 | 0.897 | 0.942 |
| 2 | 0.98 | 0.97 | 0.921 | 0.951 |
| 3 | 0.98 | 1 | 0.901 | 0.933 |
| 24h | Control | ATT | LPS | LPS+ATT |
| 1 | 0.98 | 0.99 | 0.83 | 0.901 |
| 2 | 0.97 | 0.98 | 0.84 | 0.897 |
| 3 | 0.98 | 0.97 | 0.82 | 0.882 |

Figure 5B

| | Control | LPS | LPS+Fer-1 |
|---|---|---|---|
| 1 | 0.98 | 0.845 | 0.932 |
| 2 | 0.979 | 0.832 | 0.965 |
| 3 | 0.982 | 0.851 | 0.91 |

Figure 5C WB

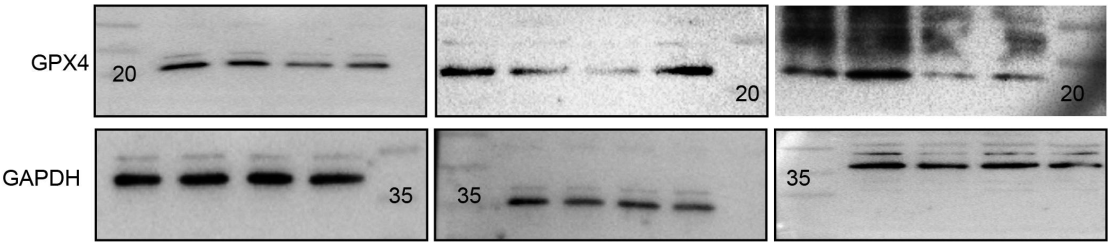


| GPX4 | Control | ATT | LPS | LPS+ATT |
|---|---|---|---|---|
| 1 | 1.011 | 1.001 | 0.632 | 0.784 |
| 2 | 0.989 | 0.987 | 0.741 | 0.851 |
| 3 | 0.992 | 1.103 | 0.605 | 0.896 |

Figure 6A

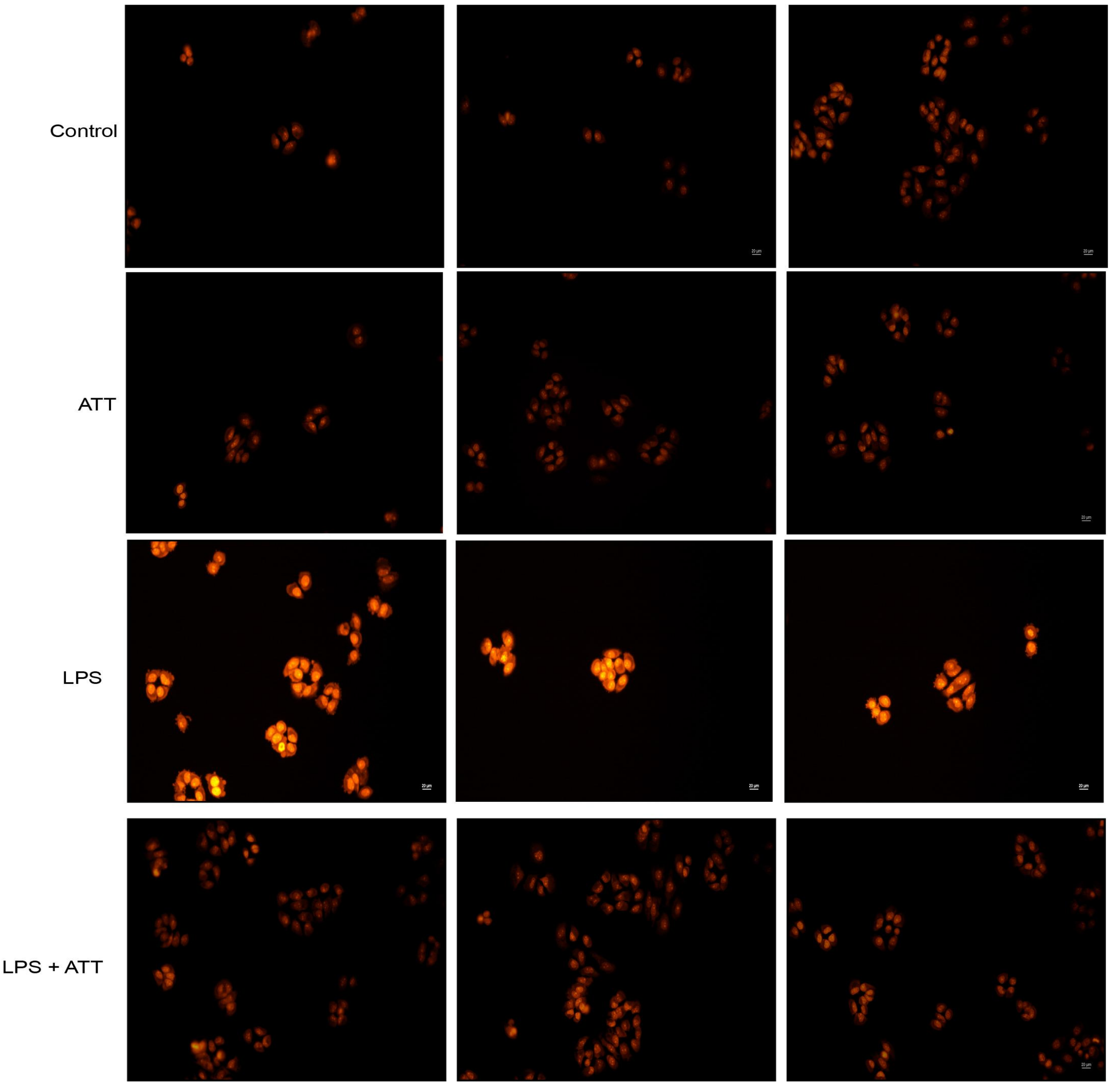

| DHE | Control | ATT | LPS | LPS+ATT |
|---|---|---|---|---|
| 1 | 1.012 | 0.832 | 3.571 | 1.523 |
| 2 | 0.987 | 0.945 | 3.674 | 1.033 |
| 3 | 0.946 | 1.013 | 4.011 | 1.215 |

Figure 6B

| SOD | Control | ATT | LPS | LPS+ATT |
|---|---|---|---|---|
| 1 | 1.019 | 1.015 | 0.455 | 0.816 |
| 2 | 0.987 | 1.024 | 0.513 | 0.799 |
| 3 | 0.976 | 0.974 | 0.462 | 0.686 |
| GSH | Control | ATT | LPS | LPS+ATT |
| 1 | 1.213 | 1.31 | 0.615 | 0.877 |
| 2 | 1.119 | 1.245 | 0.542 | 0.901 |
| 3 | 1.204 | 1.132 | 0.633 | 0.845 |
| MDA | Control | ATT | LPS | LPS+ATT |
| 1 | 0.602 | 0.645 | 1.423 | 0.998 |
| 2 | 0.713 | 0.789 | 1.323 | 1.023 |
| 3 | 0.775 | 0.692 | 1.406 | 1.116 |

Figure 6C

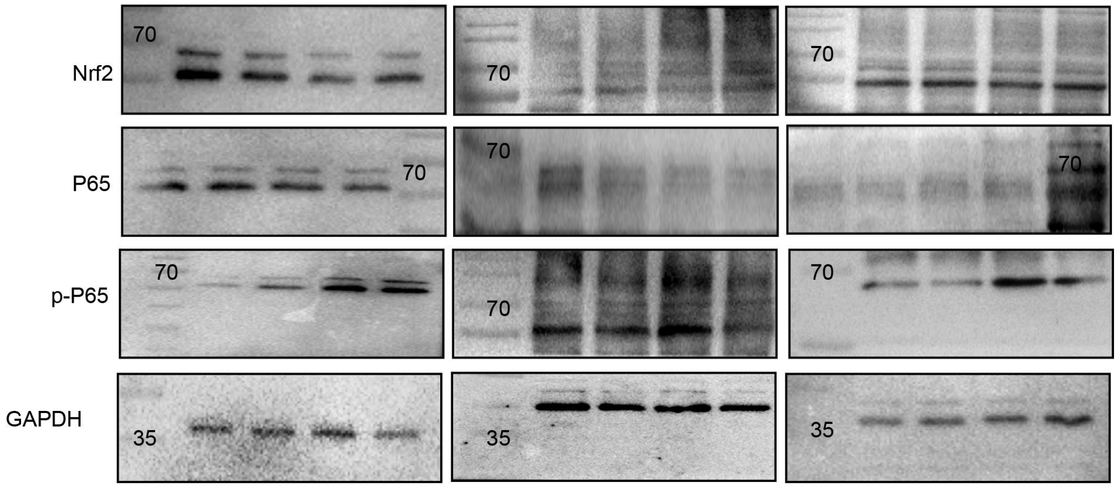


| Nrf2 | Control | ATT | LPS | LPS+ATT |
|---|---|---|---|---|
| 1 | 1.011 | 0.989 | 0.643 | 0.876 |
| 2 | 0.987 | 1.022 | 0.527 | 0.745 |
| 3 | 0.992 | 0.998 | 0.516 | 0.688 |
| p-P65 | Control | ATT | LPS | LPS+ATT |
| 1 | 1.003 | 1.101 | 3.012 | 2.102 |
| 2 | 0.986 | 0.987 | 3.244 | 2.455 |
| 3 | 0.992 | 0.969 | 2.987 | 2.01 |

Figure 6D

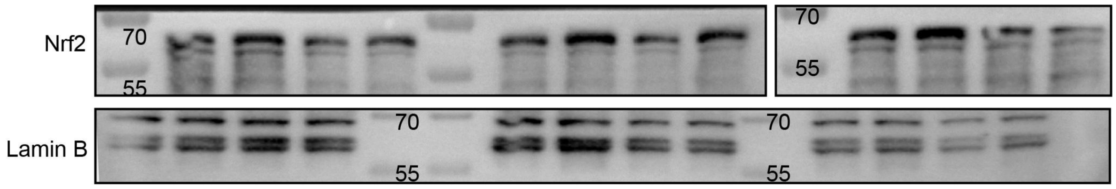


| Nu NRF2 | Control | ATT | LPS | LPS+ATT |
|---:|---:|---:|---:|---:|
| 1 | 1.011 | 1.023 | 0.764 | 0.934 |
| 2 | 0.998 | 1.011 | 0.632 | 0.876 |
| 3 | 1 | 1.015 | 0.611 | 0.921 |

Figure 6E

| MTT | Control | LPS+ATT | LPS+MI385 | LPS+MI385+ATT |
|---:|---:|---:|---:|---:|
| 1 | 1.078 | 0.987 | 0.674 | 0.813 |
| 2 | 0.998 | 0.897 | 0.655 | 0.842 |
| 3 | 1.001 | 0.942 | 0.701 | 0.767 |

Figure 6F-G

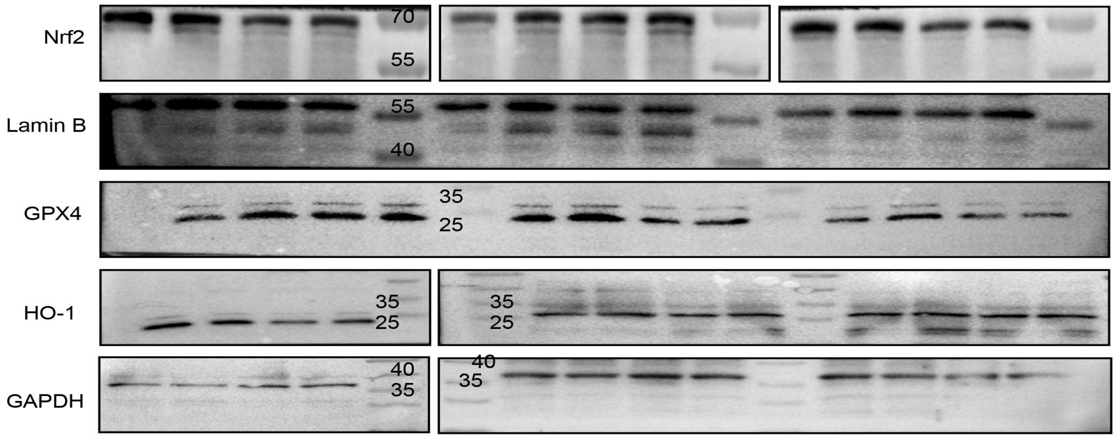


| Nu Nrf2 | Control | LPS+ATT | LPS+MI385 | LPS+MI385+ATT |
|---:|---:|---:|---:|---:|
| 1 | 1.001 | 0.987 | 0.678 | 0.876 |
| 2 | 0.978 | 0.976 | 0.632 | 0.865 |
| 3 | 1.012 | 0.899 | 0.701 | 0.742 |
| GPX4 | Control | LPS+ATT | LPS+MI385 | LPS+MI385+ATT |
| 1 | 1.013 | 1.102 | 0.732 | 0.987 |
| 2 | 0.993 | 0.989 | 0.645 | 0.976 |
| 3 | 1.005 | 1.004 | 0.598 | 0.992 |
| HO-1 | Control | LPS+ATT | LPS+MI385 | LPS+MI385+ATT |
| 1 | 1.011 | 0.988 | 0.645 | 0.986 |
| 2 | 0.998 | 0.976 | 0.567 | 0.977 |
| 3 | 1 | 1.012 | 0.543 | 0.894 |
